# Easy and non-invasive disease detection in pigs by adenosine deaminase activity determinations in saliva

**DOI:** 10.1371/journal.pone.0179299

**Published:** 2017-06-08

**Authors:** Ana María Gutiérrez, Ernesto De La Cruz-Sánchez, Ana Montes, Juan Sotillo, Cándido Gutiérrez-Panizo, Pablo Fuentes, Pedro Luis Tornel, Juan Cabezas-Herrera

**Affiliations:** 1 Department of Animal Medicine and Surgery, Regional Campus of International Excellence “Campus Mare Nostrum”, University of Murcia, Espinardo, Murcia, Spain; 2 Department of Physical Activity and Sport, Regional Campus of International Excellence “Campus Mare Nostrum”, University of Murcia, San Javier, Murcia, Spain; 3 Cefu S.A., Alhama de Murcia, Murcia, Spain; 4 Clinical Analysis Service, University Hospital Virgen de la Arrixaca, El Palmar, Murcia, Spain; 5 Molecular Therapy and Biomarkers Research Group, Clinical Analysis Service, University Hospital Virgen de la Arrixaca, IMIB-Arrixaca, El Palmar, Murcia, Spain; University of the Pacific, UNITED STATES

## Abstract

The quantification of adenosine deaminase (ADA) in porcine saliva samples has been analyzed for its use as a marker of disease. First, an analytical validation of the enzymatic assay used for ADA measurements was performed. Afterwards, saliva samples were collected from 50 healthy animals and 64 animals with different symptoms of disease, which were divided into local inflammation, gastrointestinal disorder, respiratory disorder and growth retardation. To optimize ADA measurements, total ADA (tADA), specific ADA (sADA) and ADA isoforms 1 and 2 activities were calculated. Moreover, to preliminarily estimate the diagnostic value of tADA activity measurements for disease detection, receiver operating characteristic (ROC) analyses was performed and compared to the results obtained for salivary acute phase proteins, haptoglobin (Hp) and C-reactive protein (CRP). The salivary levels of tADA activity were significantly elevated in animals with local inflammation, gastrointestinal disorder and respiratory disorder. The calculation of the different ADA activities did not provide additional information to tADA activity quantification for disease detection. The diagnostic value of tADA activity was superior to those observed for Hp and CRP measurements in the present study. It might be concluded that ADA analysis in saliva could be used as a simple, rapid, economic and non-invasive diagnostic tool in porcine production in field conditions.

## Introduction

Non-invasive sampling methodologies for health status monitoring of farm animals comply with the general objectives of the EU concerning food safety policy (Regulation 652/2014) in which animal welfare and wellbeing are major issues. Saliva offers a source of locally produced and serum-derived markers with non-invasive and minimally stressful animal practices. Several advantages for the use of saliva have been reported in pigs and have focused a great interest during the last decade in porcine, such as the efficient and low-cost collection of large numbers of diagnostic samples [1] or the possibility of performing repeated sampling without causing stress [2]. However, the reported low levels of the markers used to assess health status using saliva samples, as observed in acute phase proteins, need highly sensitive technologies for their proper quantification. Thus, searching for rapid, economic and easy assays of health markers with high diagnostic value quantification is of great interest.

Several lines of evidence have identified the adenosine system as a powerful, evolutionary selected mechanism, involved in the regulation of various processes related to inflammatory response and protection of tissues from injury. Pathological events, such as hypoxia or inflammation, result in increasing adenosine levels at an early stage of the abnormal condition [3] in order to inhibit inflammation, to protect from hypoxia- and ischemia-induced tissue damage, and to diminish the release of inflammatory cytokines. Indeed, in chronic injury, adenosine is detrimental due to the promotion of excessive tissue repair and fibrotic responses [4]. In this context, the induction of the catabolic enzyme, adenosine deaminase (ADA), represents an important checkpoint to down-regulate extracellular adenosine levels and, consequently, modulate adenosine receptor stimulation [5].

Adenosine deaminase (ADA) is an enzyme that catalyzes the adenosine removal in the purine metabolic pathway. ADA is involved in the differentiation and maturation of the immune cells including lymphocytes and monocyte-macrophage cell lines [6]. Two isoforms ADA1 and ADA2 have been reported with unique biochemical properties [7]: the isoform ADA1 exists in all human tissues, while ADA2 is the main ADA isoenzyme in serum, originated mainly from the monocyte-macrophage system [8].

Several studies have shown that circulating ADA levels are elevated in some diseases, which may represent a compensatory mechanism due to the elevated levels of adenosine and the release of inflammatory mediators. Variations in ADA activity levels have been described in different types of cancer [9] and in inflammatory diseases such as rheumatoid arthritis [10], celiac disease [11], ulcerative colitis [12], systemic lupus erythematosus [13], visceral leishmaniasis [14] and inflammatory obesity [15] or infections such as tuberculosis [16] in human serum, saliva or sputum samples. Moreover, correlations between the increases of ADA levels and other inflammatory markers such as C-reactive protein (CRP) have been reported in diseases with inflammatory components such as gestational diabetes mellitus [17] or rheumatoid arthritis [18]. However, the possible diagnostic value of ADA quantifications in animals has not been described until now. There is only one proteomics report that suggests ADA as a possible marker of disease in pigs [19].

In the present study, the quantification of the ADA levels in saliva of pigs is reported for the first time by using a properly validated commercial enzymatic assay. In addition, the diagnostic value of its quantification for the detection of disease has been analyzed in animals with different disorders (local inflammation, intestinal disorders, growth retardation and respiratory disorders). Moreover, the different ADA isoforms have been quantified and analyzed and the optimal calculation of ADA activity for porcine disease detection in field conditions using saliva samples has been proposed.

## Material and methods

### Animal sampling and classification

A total of 114 pigs conventional Duroc x (Landrace x Large White) were included in the study from four different farms located in the south east of Spain. One farm was classified as pathogen specific free and has animals of high sanitary status. The other three farms were conventional farms with commercial acceptable sanitary status. All pigs were sampled during routine veterinary field examinations at finishing stage in four different days (one day/farm). The names and geographic coordinates for the farms in which samples were taken are: Madax: 38°26'14.2"N 1°33'23.6"W, Universidad: 38°00'28.4"N 1°10'28.9"W, Camarroja: 37°48'36.2"N 1°25'08.0"W and Cuesta: 37°48'45.0"N 1°22'21.1"W.

Overall, 50 clinically healthy pigs and 64 pigs with varied signs of disease were sampled. From the first farm, 20 clinically healthy animals were randomly selected and sampled. No pigs with clinical symptoms of disease were observed in this farm during the veterinary visit. From a second farm 10 clinically healthy pigs were randomly selected along with a total of 45 pigs with diverse signs of disease. Moreover, 10 clinically healthy pigs and 7 pigs with varied respiratory symptoms were sampled from another farm subjected to a respiratory outbreak. Finally, 10 clinically healthy pigs and 12 pigs with several apparent disorders were sampled from the last conventional farm.

Management conditions related to feeding and housing were the same in all farms since all farms belongs to the same large commercial pig company. The average number of growing pigs per farm was around 4000 animals. Pigs were housed in pens in groups of 10 animals with a minimum of 0.65 square meters per animal (Directive 2001/88/CE). All pigs were given ad libitum access to a nutritionally balanced commercial diet (3300 kcal ED/kg, 3.1% fiber, 16% protein, and 5% fat) and water was continuously available. All procedures involving animals were approved by the Murcia University Ethics Committee and followed the recommendations of the European Convention for the Protection of Vertebrate Animals used for Experimental and Other Scientific Purposes (Council of Europe, ETS no. 123). All methods were performed in accordance with the relevant ARRIVE guidelines and regulations.

Sampling procedure consisted in allowing the pigs to chew a sponge measuring approximately 1 cm x 1 cm in size, clipped to a thin metal rod, for 1–2 minutes as previously reported [20]. Individual saliva collection in pigs was performed without picking up the animals. Saliva was obtained by centrifugation of sponges inserted in specific tubes (Salivette tubes, Sarstedt, Nümbrecht, Germany) for 10 min at 3000 x g. Saliva was collected from all animals in the early morning at the farms. The term “saliva” in the whole study refers to a complex body fluid composed by a mixture of salivary and non-salivary components as reported before [2].

All animals were subjected to a general clinical veterinary examination at the farms. The parameters taken into account during the examination were detailed observation of the patient and the other pigs in the group and their environment, the general aspect of the individual animals and annotation of any clinical sign of disease or abnormal behaviour. Afterwards, animals were classified into five groups depending on the signs of disease observed during the veterinary examination as: clinically healthy animals (CH, n = 50), animals with local inflammation (LI, n = 15), gastrointestinal disorders (GD, n = 14), growth retardation (GR, n = 19) and respiratory diseases (RD, n = 16). All animals classified into each group presented one or more of the clinical manifestations considered for the corresponding group as detailed in Table 1. Animals classified into the CH group presented a good body condition and skin colour with no apparent symptoms of any disorder and a normal behaviour during the veterinary clinical examination. Moreover, for overall objective inflammatory-infection condition assessment and to identify any possible subclinical disorder, two acute phase proteins were quantified in saliva samples of all animals, specifically haptoglobin (Hp) and C-reactive protein (CRP). For Hp and CRP quantifications, previously developed and validated assays were used [20, 21].

**Table 1:** Main signs of disease / symptoms observed in the different groups of animals during the clinical veterinary examination.

Animal group	Clinical manifestations
Healthy	• No clinical signs of disease
Local inflammation	• Superficial lumps or wounds • Joint swelling • Limp • Tail-biting signs • Exudative epidermitis
Gastrointestinal disorder	• Intestinal hernia • Rectal prolapse • Sick • Diarrhea
Growth Retardation	• Retardation in grow rate • Dull skin and hair • Rough hair coat
Respiratory disorder	• Cough • Nasal secretion • Sneeze • Dyspnea • Fatigue • Cyanosis

### Measurements of ADA activity levels

ADA activity levels in saliva samples were measured by using an adaptation to microtritation plates of a commercial automatized assay (BioSystems S.A., Barcelona, Spain) used for human ADA determinations in pleural fluid. The adaptation consisted of the use of 50μL of diluted saliva and 200μL of ADA reagent (prepared according to manufacturer’s instructions). The method is based in the deamination of adenosine to inosine by adenosine deaminase. The catalytic concentration is estimated by the oxidation of NADH to NAD+ in a coupled reaction:

$$Adenosine+H_{2}O\to Inosine+{NH}_{4}^{+}$$

$$2-Oxoglutarate+{NH}_{4}^{+}+NADH+H^{+}\to Glutamate+{NAD}^{+}$$

The reaction was measured at 340nm in a kinetic mode and the maximal increase in absorbance per minute was recorded. The levels of total ADA activity (ADA) were calculated in U/L according to manufacturer’s instructions (ΔA/min x 3333 = U/L).

Three different ADA activity measurements were calculated, total ADA (tADA) activity, specific ADA (sADA) activity and ADA isoforms 1 and 2 (ADA1 & ADA2, respectively) activities. For the calculation of the sADA activity, which represents the enzyme activity per milligram of total proteins, the concentration of the total protein of each saliva sample was measured according to Bradford [22]. Moreover, to determine ADA2, ADA1 activity was selectively inhibited by the addition of erythro-9-(2-hydroxy-3-nonyl) adenine (EHNA) into the saliva samples and estimated ADA1 was calculated by subtracting ADA2 from tADA activity.

### Analytical validation of ADA activity assay

Prior to the analytical validation study, the optimal saliva sample dilution of use in the ADA assay was calculated by investigating the saliva dilution that gives the higher absorbance level without hook effect and initial interferences. The analytical validation study consisted of the analysis of several parameters including precision, accuracy and limit of detection as performed before for other assays [20, 21].

For precision analysis intra-assay variations were investigated by the calculation of the coefficient of variation of the measurement of eight replicates of two pools of saliva samples of high, medium and low ADA activity levels in the same analytical run. Accuracy was evaluated indirectly by linear regression analysis of serial saliva dilutions from two samples with high levels of ADA activity.

The limit of detection was analyzed by measuring ten replicates of a zero blank sample in the same analytical run.

The stability of tADA activity levels in saliva samples stored at -80°C was analyzed in a period of 7 months. Three batches of saliva samples were prepared for high, medium and low tADA activity levels. Each batch was composed of 5 saliva samples of similar tADA activity levels and was analyzed at 0, 4, 5 and 7 months of storage at -80°C.

### Overlap performance and proof of concept

The possible application of the measurements of tADA activity levels for disease detection in pigs was assessed by investigating the levels of tADA activity in clinically healthy animals and in animals with different clinical symptoms of disease of different origins. Moreover, the same analysis was repeated for Hp and CRP quantifications, and the results from the three markers, tADA, Hp and CRP, were compared.

Saliva samples from a total of 50 clinically healthy animals and 64 pigs with several signs of disease were quantified for tADA activity, Hp and CRP levels.

A time-course analysis was performed as a proof of concept. ADA levels were monitored in diseased animals after treatment. For that end, five animals with severe clinical symptoms of respiratory disease such as fatigue, dyspnea and prostration, were selected from the second farm and subjected to a common antibiotic treatment. Treatment consisted on individual antibiotic therapy with two intramuscular injections of florfenicol at 15 mg/kg separated 48 hours. Selected animals were sampled for saliva at different days, at the beginning of the study just before treatment (T0) and one (T1), two (T2) and four (T4) days after treatment and the tADA activity levels, were quantified in all time-points.

### Statistical analysis

All statistical analyses were carried out using statistical software (GraphPad Prism 5; GraphPad software Inc., Suite, La Jolla, USA).

For the precision analysis, the coefficient of variation (%) was calculated by the standard deviation divided by the mean per 100. The linear regression construction used to indirectly calculate the accuracy of the assay was estimated by the comparison of expected and observed results and the calculation of the regression coefficient (R square, as a goodness of fit measure). The limit of detection was calculated as the mean plus two times the standard deviation of the measurements. Finally, to establish the stability of tADA measurements during storage at -80°C, the percentage of variation of each measurement in comparison to the basal level was calculated. Changes that exceeded ± 2 intra-assay coefficients of variation of the assay and had significant (P < 0.05) differences over time were considered to indicate unacceptable stability for given storage conditions as reported before [23].

The additional value of each ADA activity measurement, sADA and ADA1 and ADA2, to tADA was analyzed by searching for differences in its capacity to distinguish between clinically healthy animals and pigs with different symptoms of disease. Moreover, the correlations between the different ADA activities calculations obtained were determined by nonparametric Spearman correlation analysis. Afterwards, according to the results, tADA activity was selected for further analysis.

Overall, Hp, CRP and tADA activity quantifications did not meet the normal distribution criteria according to a Kolmogorov-Smirnov test, so non-parametric tests were used for statistical comparisons. tADA activity, Hp and CRP levels in the different groups of animals were compared by using a Kruskal-Wallis test with Dunn’s multicomparisons test. Afterwards, the correlation between the different parameters measured, tADA, Hp and CRP, were determined by nonparametric Spearman correlation analysis.

Receiver operating characteristic (ROC) analyses was performed in order to determine, as a first approach, the threshold laboratory value of each analyte, which separates a clinical diagnosis of “healthy” from one of “diseased”. The areas under the curves for Hp, CRP and tADA activity were obtained to quantify the overall ability of each test to discriminate between diseased and non-diseased pigs.

A Friedman test was run to determine if there were differences in tADA concentrations during the time course analysis used as proof of concept. Pairwise comparisons were performed with a Dunn’s correction for multiple comparisons.

## Results

### ADA activity assay validation

**Fig 1:**
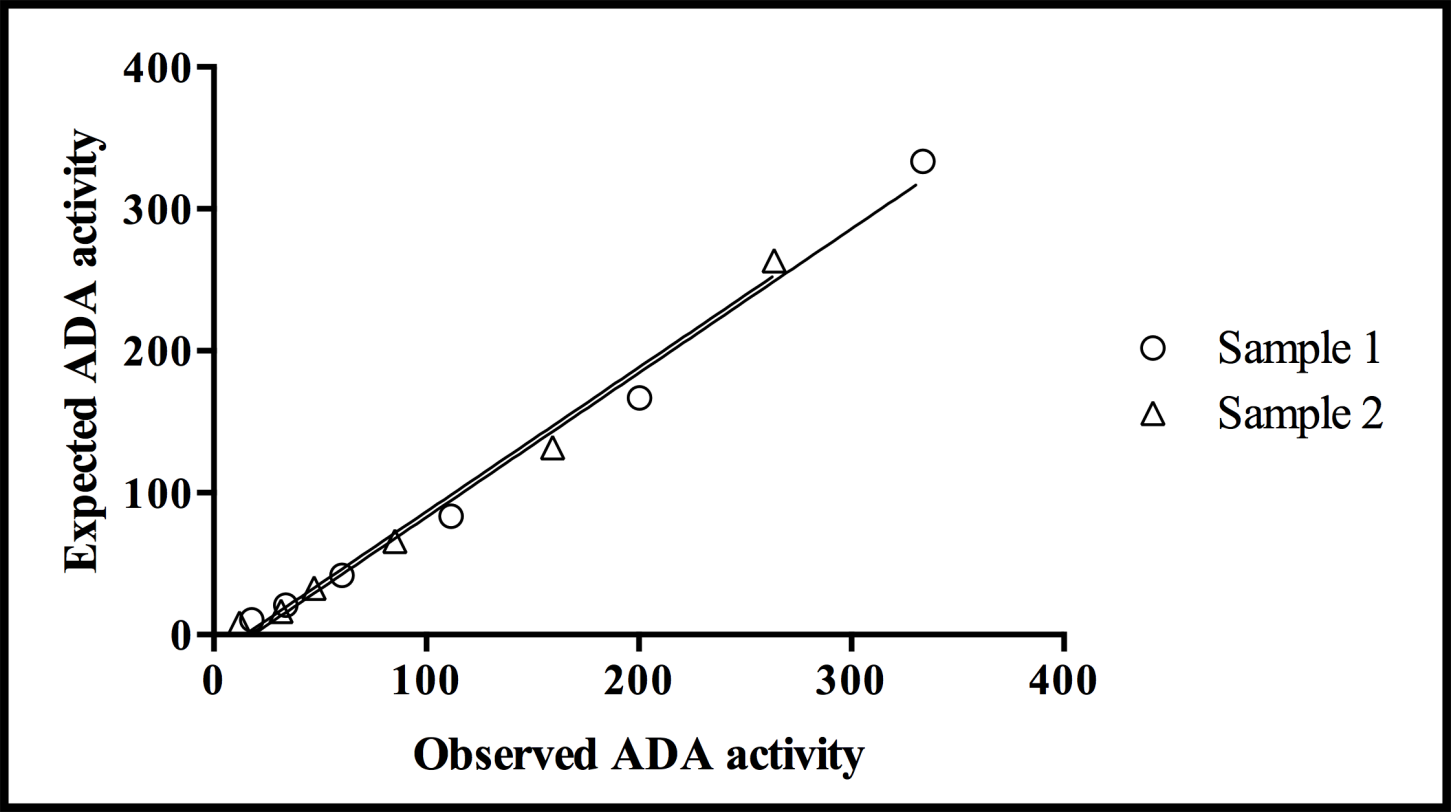
Linear regression lines indicating the accuracy of the enzymatic assay used for measuring ADA activity in serial dilutions of 2 samples of saliva from swine. The slope of the regression lines for both samples (circle and triangle symbols) was 1.016 and the intercept was 18.49 (*R*^2^ = 0.98).

The overall coefficients of variation obtained in the precision analysis were lower than 6% for high, medium and low tADA activity levels (Table 2). Moreover, the coefficients of correlation observed in the linear regression study were close to 1, specifically 0.989 in the two samples studied (Fig 1), and the limit of detection calculated for the ADA activity assay under the adapted conditions was 9.33 U/L.

**Table 2:** Intra-assay coefficient of variation for ADA activity assay.

	X	SD	CV
**High ADA levels**	245,13	13,34	5,44
**Medium ADA levels**	94,07	3,12	3,32
**Low ADA levels**	32,32	1,74	5,39

X = median (UI/L). SD = standard deviation. CV = coefficient of variation (%).

**Fig 2:**
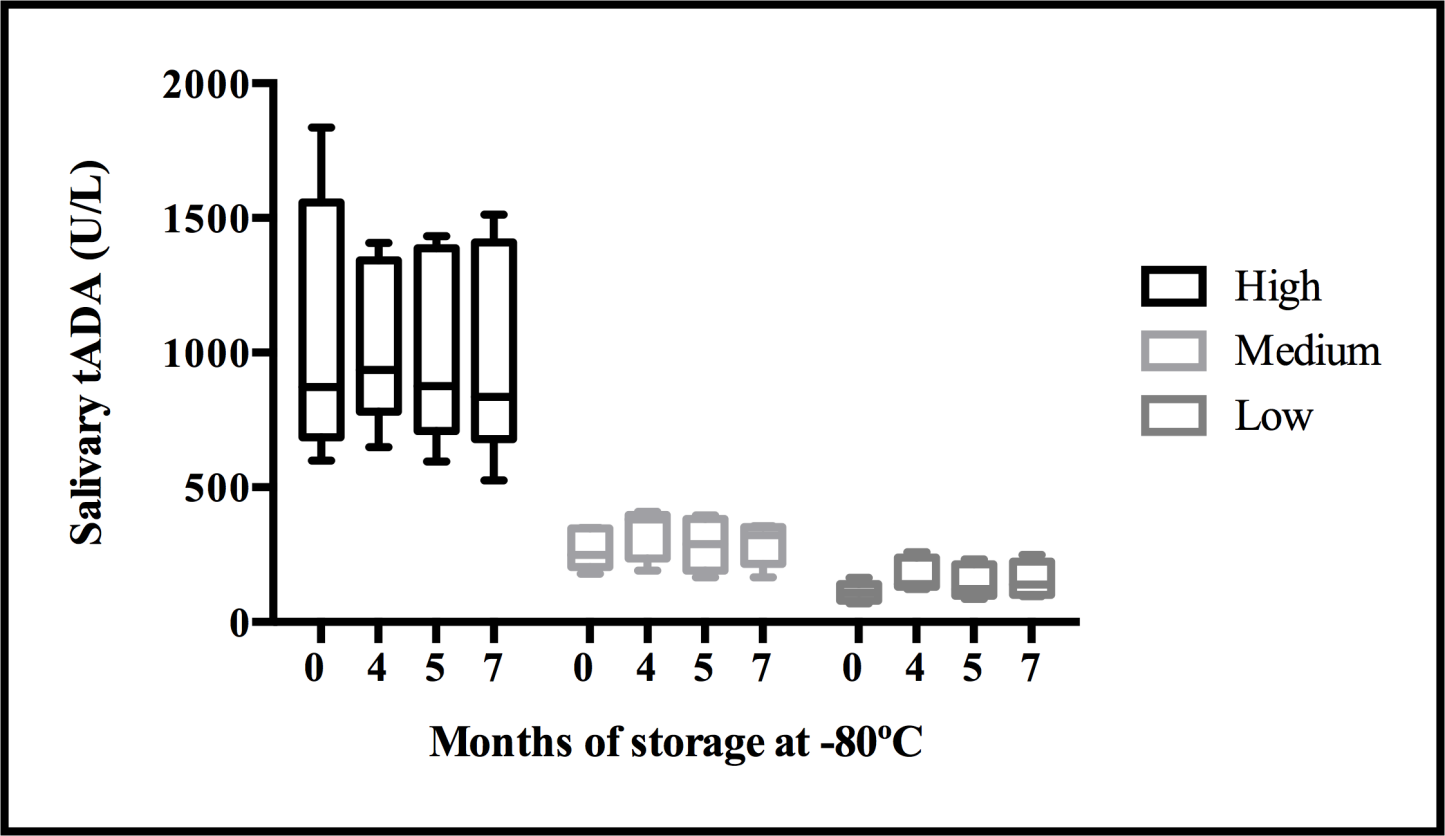
Measurement of ADA activity, in pooled saliva samples of high, medium and low levels, during storage of 7 months at -80°C. The plot shows median (line within box), 25th and 75th percentiles (box), 5th and 95th percentiles (whiskers).

The study of the tADA activity measurement stability in saliva samples stored at -80°C during a period of 7 months showed no statistical significant differences with the basal measurements (Fig 2). Moreover, the mean coefficients of variation of the measurements of the saliva samples of high, medium and low tADA activity content was lower than 8% in the whole stability period analyzed.

### Overlap performance and proof of concept

Three different ADA activity measurements were obtained, tADA activity, sADA activity and ADA1 and ADA2 activity, with different correlations between each other (Table 3). The higher correlations coefficients were observed between tADA and ADA2 activity quantifications.

**Table 3:** Spearman coefficients of correlation (r) between the different ADA results, total ADA activity (tADA), isoforms ADA1 and 2 (ADA1 & ADA2) and specific ADA activity (sADA).

	ADA2	ADA1	sADA
tADA	0,852**	0,761**	0,378**
ADA2		0,388**	0,206*
ADA1			0,428**

*level of significance P < 0.05.

**level of significance P < 0.001.

**Fig 3:**
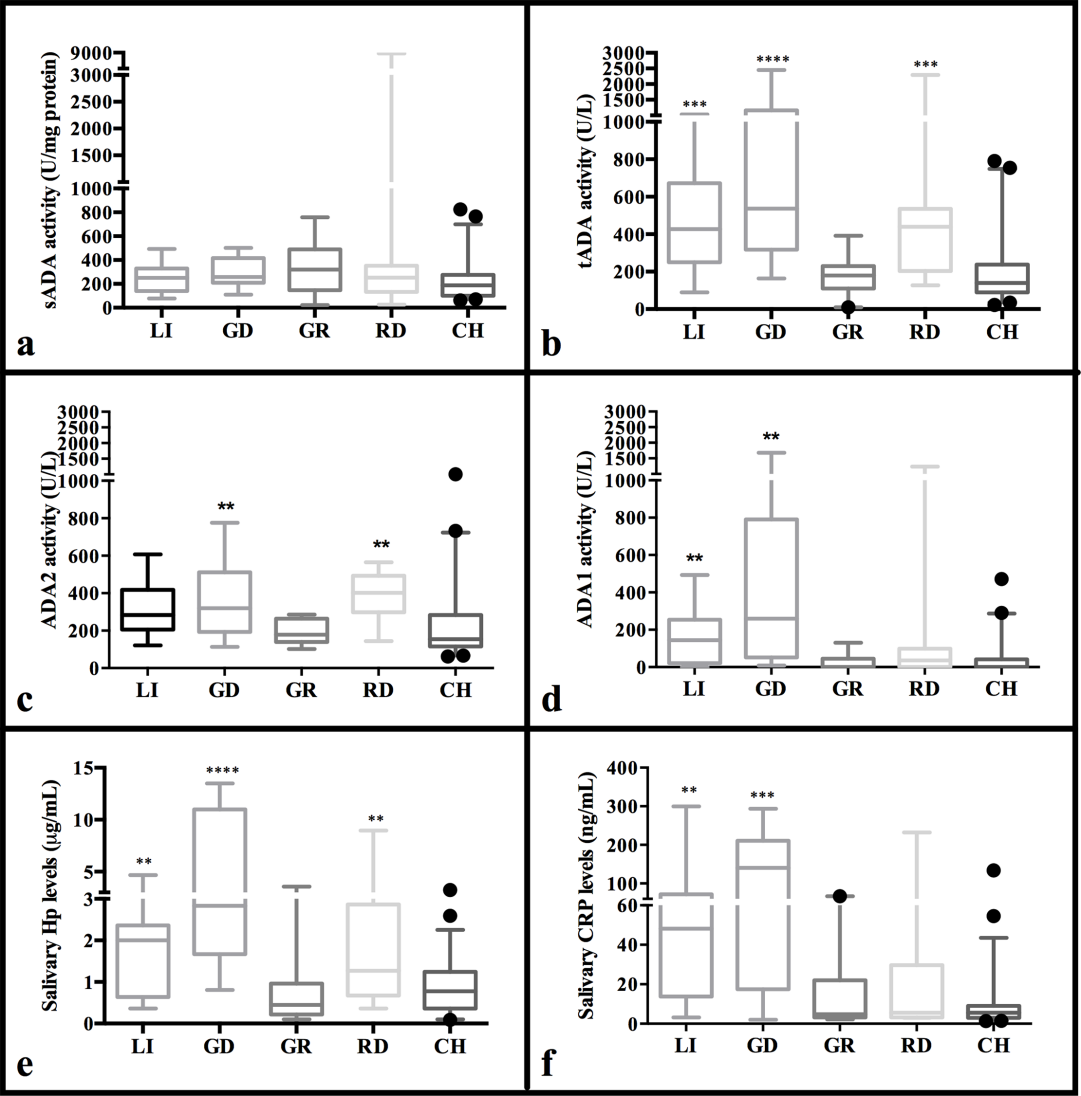
Median concentrations of adenosine deaminase and acute phase proteins measurements in a group of clinically healthy animals (CH, n = 50) and in animals with local inflammation (LI, n = 15), gastrointestinal disorders (GD, n = 14), growth retardation (GR, n = 19) and respiratory diseases (RD, n = 16). Results expressed as sADA (a), tADA (b), ADA2 (c), ADA1 (d), Hp (e) and CRP (f). The plot shows median (line within box), 25th and 75th percentiles (box), 5th and 95th percentiles (whiskers) and outliers (•). Asterisk represents the statistically significant differences from each group: **level of significance P < 0.01. ***level of significance P < 0.01. ****level of significance P < 0.001.

The differences observed in activity between the different groups of animals vary depending on the ADA activity measured since sADA activity values did not show any statistical significant differences between the values in healthy animals and the rest of groups of animals with signs of disease (Fig 3A), while tADA activity quantifications showed several differences. The median levels of tADA activity in healthy pigs were 140 U/L (88.94 and 237 U/L as 25^th^ and 75^th^ percentiles respectively), similar to those observed in animals with growth retardation (180 U/L, 110 and 230 U/L as 25^th^ and 75^th^ percentiles respectively), and statistically significantly higher than those observed in the groups of animals with local inflammation (426.6 U/L, 250 and 672.2 U/Las 25^th^ and 75^th^ percentiles respectively), gastrointestinal disorders (536.6 U/L, 318 and 1160 U/L as 25^th^ and 75^th^ percentiles respectively) and respiratory diseases (440.1 U/L, 203.6 and 535 U/L as 25^th^ and 75^th^ percentiles respectively) (Fig 3B). Although the calculation of ADA1 and ADA2 did not incorporate any additional information to tADA activity, both isoforms showed statistical significant differences between groups. Specifically, ADA1 activity in healthy animals was statistically significantly lower than the values observed in pigs suffering from local inflammation and gastrointestinal disorders (Fig 3C). Moreover, the levels of ADA2 activity observed in animals suffering from gastrointestinal and respiratory disorders were statistically significantly higher than those reported in healthy animals (Fig 3D).

Overall, the levels of acute phase proteins, Hp and CRP, in healthy animals were statistically significantly lower than those observed in animals with signs of disease (p < 0.0001). However the differences in the levels quantified in the groups of animals with clinical symptoms of disease in comparison to clinically healthy animals differ between markers. The median concentration of Hp in the group of healthy animals was 0.77 μg/mL (0.36 and 1.24 μg/mL as 25^th^ and 75^th^ percentiles respectively). Statistical significant differences were obtained with several groups of symptomatic animals (Fig 3E), specifically with local inflammation (p = 0.0031; median value = 2 μg/mL (0.64 and 2.36 μg/mL as 25^th^ and 75^th^ percentiles respectively)), gastrointestinal disorder (p < 0.0001; median value = 2.83 μg/mL (1.67 and 10.99 μg/mL as 25^th^ and 75^th^ percentiles respectively)) and respiratory disease (p = 0.0090; median value = 1.26 μg/mL (0.67 and 2.86 μg/mL as 25^th^ and 75^th^ percentiles respectively)), with lower values observed in healthy animals. Meanwhile the concentrations of Hp in the group of animals with growth retardation were lower than those reported in clinically healthy animals with a median value of 0.45 μg/mL (0.22 and 0.96 μg/mL as 25^th^ and 75^th^ percentiles respectively).

CRP levels in the group of clinically healthy pigs (median value = 4.69 ng/mL (3.02 and 9 ng/mL as 25^th^ and 75^th^ percentiles respectively)) were statistically significantly lower than those observed in the subgroups of local inflammation (p < 0.001; median value = 48.13 ng/mL (13.79 and 71.75 ng/mL as 25^th^ and 75^th^ percentiles respectively)) and gastrointestinal disorders (p < 0.0001; mean value = 140.4 ng/mL (17.41 and 210.9 ng/mL as 25^th^ and 75^th^ percentiles respectively)) (Fig 3F). No differences were observed between the concentrations of CRP in the subgroups of animals suffering from respiratory diseases or growth retardation.

**Fig 4:**
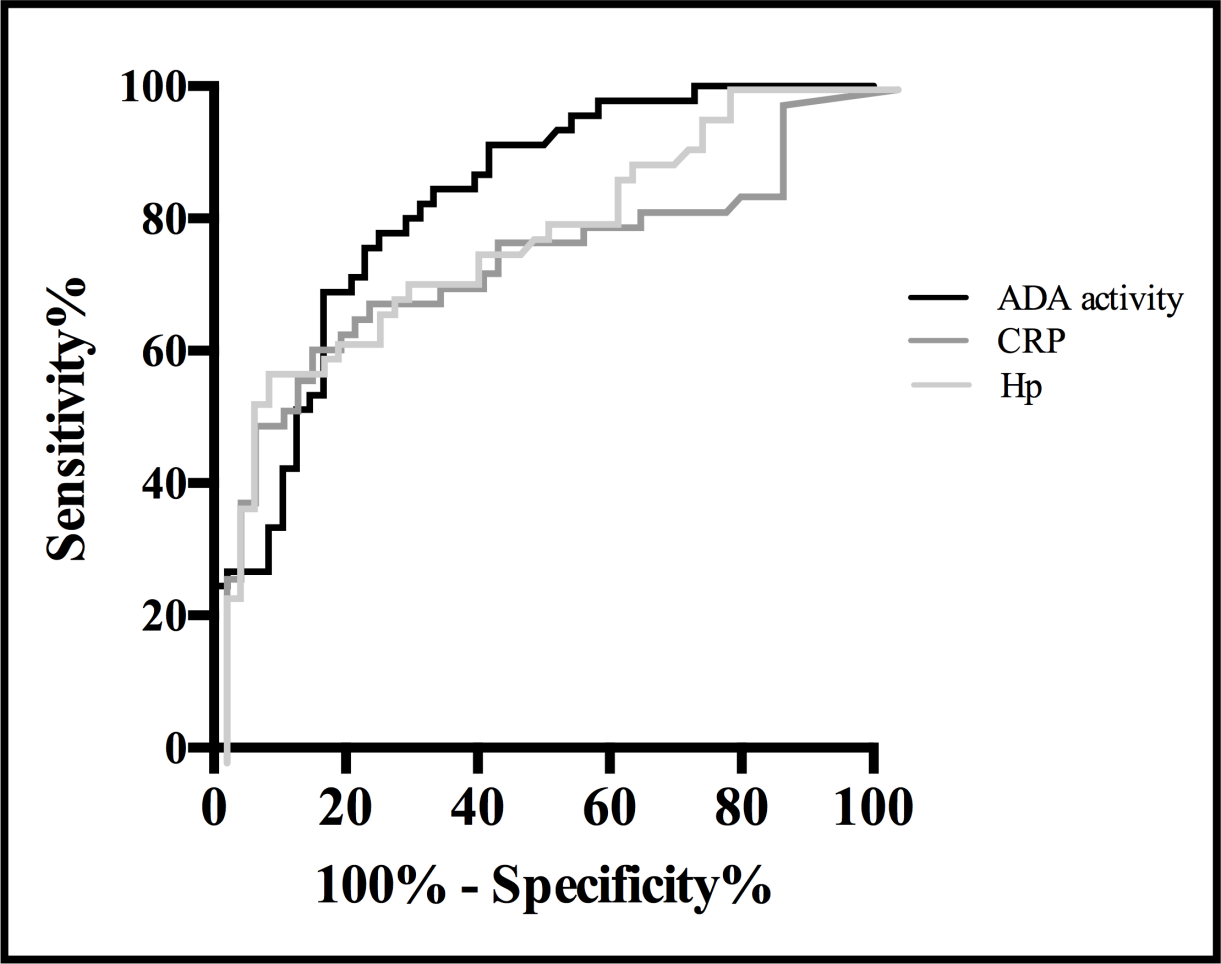
ROC curves for Hp, CRP and ADA activity to distinguish between healthy pigs and pigs with any clinical sign of disease. The curves were constructed using the data of 51 healthy pigs and 45 pigs suffering from local inflammation or gastrointestinal or respiratory disorders.

The correlation analysis performed between the quantifications of the three different biomarkers studied showed positive Spearman’s coefficients of correlation of r = 0.565 (p < 0.0001) for the correlation Hp-tADA, r = 0.535 (p < 0.0001) for the correlation Hp-CRP and r = 0.344 (p = 0.001) for the correlation CRP-tADA. Moreover, according to the results of the ROC analysis (Table 4), the AUC for tADA (0.823) was superior to the values obtained in Hp (0.789) and CRP (0.755) giving a better ROC curve (Fig 4).

**Table 4:** Overall diagnostic value comparison of Hp, CRP and tADA activity quantifications for disease detection in saliva samples of pigs.

	tADA activity	Hp	CRP
Area under the curve (AUC)	0.823	0.789	0.755
Std. Error	0.042	0.046	0.052
95% confidence interval	0.739 to 0.907	0.698 to 0.881	0.651 to 0.859
p value	< 0.0001	< 0.0001	< 0.0001

**Fig 5:**
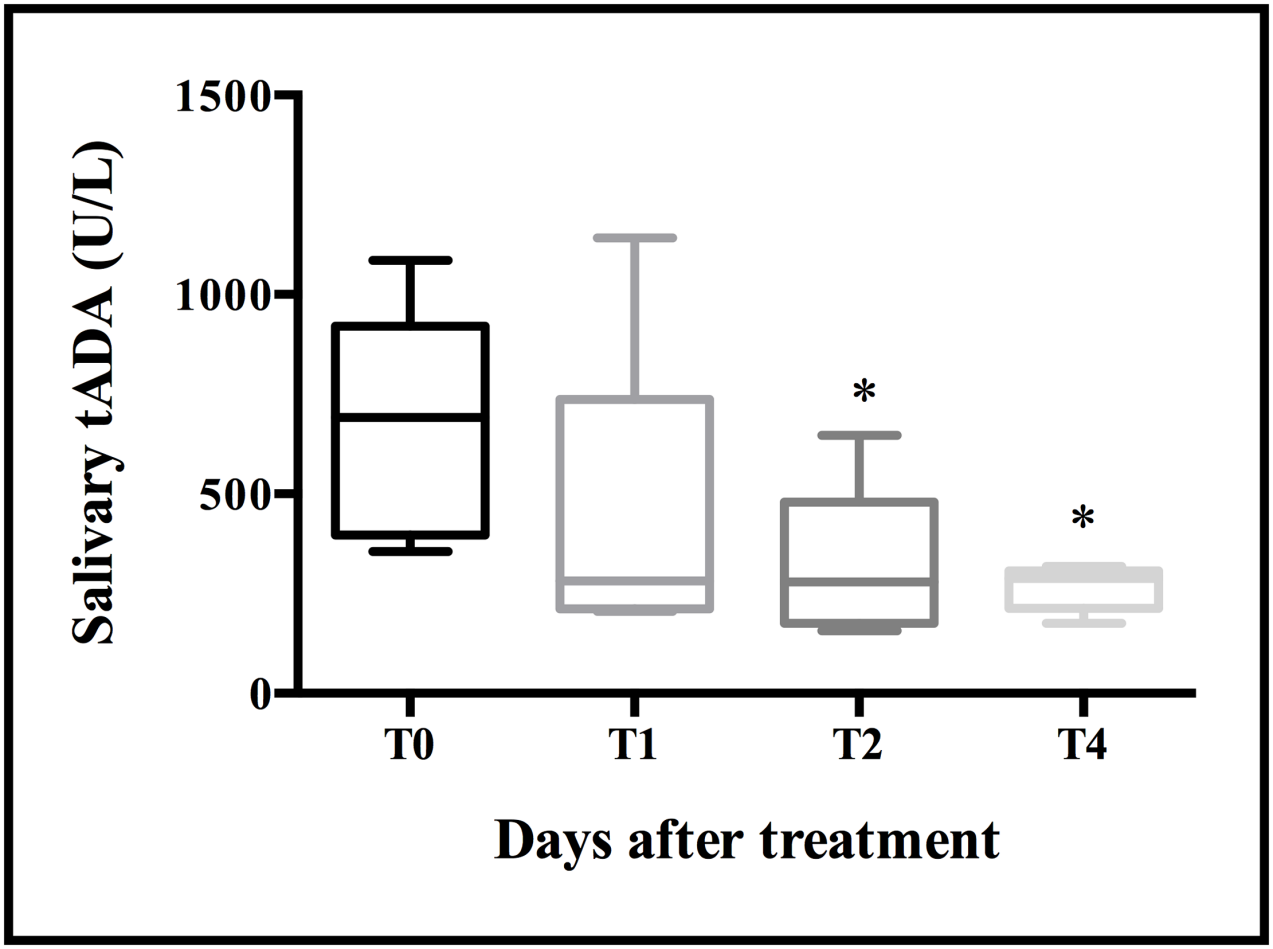
Median concentrations of total adenosine deaminase activity levels in a group of diseased animals, with severe respiratory disorders, after treatment (n = 5). The plot shows median (line within box), 25th and 75th percentiles (box), 5th and 95th percentiles (whiskers) and outliers (•). Asterisk represents the statistically significant differences: *level of significance P < 0.05.

The levels of tADA activity decrease significantly after two and four days of antibiotic treatment in animals with severe respiratory disease (Fig 5). The median levels of tADA at T0 was 691.9 U/L (396.6 and 921.2 U/L as 25^th^ and 75^th^ percentiles respectively), while the values after treatment were lower at all time-points, 282 U/L (211.6 and 736.3 U/L as 25^th^ and 75^th^ percentiles respectively) at T1, 279.3 U/L (175.3 and 479.3 U/L as 25^th^ and 75^th^ percentiles respectively) at T2 and 287.9 U/L (213.9 and 307 U/L as 25^th^ and 75^th^ percentiles respectively) at T4.

## Discussion

Our results proposed that ADA activity measured in porcine saliva samples might be a useful biomarker for disease detection. Measurement of salivary ADA activity as a simple, rapid and inexpensive diagnostic marker makes it a useful tool for health status monitoring in porcine farms using non-invasive sampling procedures.

ADA is a polymorphic enzyme that is involved in the purine metabolism since it catalyzes deamination of adenosine and deoxyadenosine [7]. A reduction in ADA activity could contribute to restrict the inflammatory response and subsequent cellular damage because it would increase adenosine tissue levels, an anti-inflammatory molecule [24]. The measurement of its activity levels in serum samples has been proved to be of great diagnostic value in several inflammatory disorders in humans; however its usefulness in animal medicine is still limited.

To study the diagnostic value of tADA activity measurement, the four most common disorders observed in the porcine production system (local inflammatory, gastrointestinal, respiratory illness and growth retardation) were monitored in a total of 64 pigs and compared to 50 healthy pigs, after a previous analytical validation of a commercially available assay. To add a useful interpretation in the field, results were compared to the quantification of two well-established acute phase proteins in saliva samples, Hp and CRP.

The optimized assay used for ADA activity measurement could be considered as precise and accurate according to the analytical validation parameters studied. The limit of detection was low enough to ensure the optimal quantification of the low levels of tADA activity observed in healthy animals. Moreover since the repeated ADA measurements performed in pooled saliva samples until 7 months, with repeated freezing and thawing, showed good stability at storage temperatures of -80°C, rapid analysis of tADA levels is not required.

It could be considered that the two isoforms of ADA, ADA1 and ADA2, are acting independently in saliva of the disorders studied in the present work since a very low correlation was observed between the different isoforms, as reported before in human serum studies [13]. Moreover, the diagnostic value of tADA could mainly be attributed to ADA2 according to the higher positive correlation observed between tADA and ADA2, similar to human serum studies [25]. On the other hand, sADA could also be considered as a separate diagnostic parameter, since no correlation was observed with tADA. However, the diagnostic value of sADA could be estimated as low or null since the measurement of sADA activity levels did not allow to distinguish between healthy animals and animals with any of the disorders mentioned above.

All the inflammatory markers quantified, ADA, Hp and CRP, were higher in symptomatic animals in comparison to clinically healthy ones, while animals with retardation in growth rate showed similar levels to those observed in healthy animals, which could be interpreted as a lack of inflammatory activation in this group. Further studies should be necessary to elucidate the mechanism behind the growth retardation condition. The salivary median levels of Hp and CRP observed in healthy animals were similar to those reported in previous studies [20, 21]. To the authors’ knowledge, this is the first time in which the levels of ADA activity have been measured in animal saliva, so the proposed values of ADA activity in healthy animals could be considered as reference values for further studies. Moreover, the time course of the salivary ADA increase still remains undefined and should be properly study. Porcine salivary ADA activity levels in healthy animals are 23 times higher than those reported for healthy humans [9]. However, several factors such as species, sex, age and the different ADA assays used could be influencing the results and should be studied in detail in the future.

The superior diagnostic value observed, in the present study, in salivary tADA levels in comparison to acute phase proteins quantifications for disease detection has been reported before in studies in which human serum ADA were recommended as a marker of inflammation in patients with rheumatoid arthritis [18] and ulcerative colitis [12].

As this is the first time in which the salivary levels of ADA activity is reported, a time course analysis was considered necessary as a proof of concept. For that end, animals with a severe respiratory disorder were selected and treated individually to study if the tADA levels decrease after treatment as a sign of recovery. Effectively the data of the analysis support the hypothesis since the tADA activity levels decrease significantly after two days of treatment raising levels similar to those obtained in clinically healthy animals in the present study.

It could be concluded that, among the other analytical tools reported until now for health status monitoring using saliva, such as measurement of acute phase proteins, ADA activity quantifications improves the prediction of disease diagnosis, since higher AUC were reported in the ROC analyses. Consequently, the authors recommend the quantification of ADA activity as a superior alternative to acute phase proteins for porcine health status assessment in the field using saliva samples.

Authors estimate that the cost of tADA test per sample could be around 1€ according to the actual available official cost of the reagents. So the benefit of using a test for ADA for either field veterinarians or farmers is based on the possibility of monitoring health status at any time by using an objective, simple and economical analytical tool to make prevention of disease a more flexible and useful instrument in the day-to-day management of the farm.

## Supporting information

S1 File Supplementary data. (XLSX)
